# Case report on the legal assurance of Advance Care Planning in collective culture

**DOI:** 10.1002/ccr3.5759

**Published:** 2022-04-20

**Authors:** Hirotomo Miyatake, Akihiko Ozaki, Yasuhiro Kotera, Ryo Sakamoto, Divya Bhandari, Yu Uneno, Hiroyuki Beniya

**Affiliations:** ^1^ Orange Home‐Care Clinic Fukui Japan; ^2^ Department of Breast Surgery Jyoban Hospital of Tokiwa Foundation Iwaki Japan; ^3^ School of Health Sciences University of Nottingham Nottingham UK; ^4^ Visina Home Care Tokyo Japan; ^5^ Medical Governance Research Institute Tokyo Japan; ^6^ Department of Therapeutic Oncology Graduate School of Medicine Kyoto University Kyoto Japan

**Keywords:** ACP, culture, home care, legal assurance

## Abstract

This case report shows that there is a lack of a legal framework in Japan to protect patients' right during the end‐of‐life period, which hinders the implementation of ACP in medical practice. This report suggests that legal support can contribute to the advancement of ACP while addressing cultural differences.

## Abstract 2

Advance Care Planning has been widely advocated in Japan, but it has not yet taken root. The collective decision making culture in Japan may unconsciously overpower or ignore the patients' wishes, hence legal support can mitigate its negative impacts on ACP.

## 1 INTRODUCTION

Progress in medicine and public health measures has led to an increase in aging population globally, and the number of people over 80 years old will be three times that of 2015 by 2050.^1^ This has resulted in an increase in chronic diseases and comorbidities.^2^ As a result, choices of treatments have become increasingly complex, making it difficult for the patients and their families to understand its details.^1^ Consequently, supporting patients in making important decisions relating to treatment choices has become even more challenging, and this is particularly true for end‐of‐life periods since it sometimes involves ethical dilemmas, such as terminating life‐sustaining treatments.^1^

In recent years, Advance Care Planning (ACP) has become a popular practice as it enables patients and their families to make a timely decision about future treatment options. In 1960s, there was a series of criticisms of medical paternalism in the United States and movements to clarify living wills and to make Advance Directive (AD).^3^ Accordingly, in 1990, the Patient Self‐Determination Act was enacted in the United States, requiring hospitals to provide information about AD and to inform patients of their right to receive or refuse treatment.^3^ Although AD was considered suitable, it proved challenging to implement in practice as patients' wishes tend to change over time.^3^ Therefore, ACP that emphasizes discussion of the desired treatment rather than AD was advocated. Recent studies have shown that ACP improves the quality of end‐of‐life care and satisfaction of patients and families, reduces stress, anxiety, depression in bereaved families,^4^ and decreases the frequency of hospitalizations,^5^ highlighting its efficiency and effectiveness in supporting patient decision‐making.

In Japan, studies on the decision‐making process for medical treatment and end‐of‐life care had been conducted since 1987,^6^ and the incident of the removal of a ventilator for euthanasia at the Imizu Municipal Hospital (Imizu City, Toyama Prefecture, Japan) in 2006 resulted in rapid progress on the decision‐making for medical treatment and end‐of‐life care in recent years.^7^ Subsequently, “Guideline for the Decision‐Making Process for Terminal Care” was formulated in 2007.^8^ The guideline includes the following points: (1) appropriate information and explanations about end‐of‐life medical care should be provided by doctors and other medical professionals, (2) patients should consult with medical professionals before making any decisions, and (3) decisions regarding medical treatment and end‐of‐life care should be made carefully by the medical and care team, not by doctors on their own. Furthermore, the guideline was revised as “Guideline for the Decision‐Making Process for Medical treatment in the end‐of‐life” in 2018, emphasizing the importance of ACP approach by the statement that individual's wishes can change according to changes in physical and psychological conditions, policy of medical treatment and care, and the patient's willingness to live, etc., which need to be discussed on a daily basis.^9^, ^10^ Furthermore, the Ministry of Health, Labor and Welfare (MHLW) commissioned the "Project for Improving the Medical System in the End‐of‐life and Education for Implementing End‐of‐Life Discussion" (E‐FIELD).^11^ This is an educational project for medical professionals to promote ACP, which has been conducted nationwide to further promote and implement ACP in the medical field.^10^ However, since ACP was originally developed in the United States and Europe with proper legal support,^12^ the implementation of ACP in Japan without such legal backing has unique challenges, and solutions to these challenges need to be considered. We have presented a case study that shows how the lack of strict legal guidance can cause a problem for the medical team to follow the patient's desire for the end‐of‐life care.

## 2 CASE

A 97‐year‐old man living with his eldest daughter and her family was a patient with hypertension and chronic kidney disease who used to seek regular treatment from a general hospital. Suddenly in January 2016, he visited the emergency department with a complaint of urinary incontinence, frequent urination, and constipation for the first time. Sennoside tablet 12 mg was prescribed for his constipation; however, his symptoms did not ease even after 3 days of administrating medicine. Thus, at the request of his elder daughter, the visiting doctor visited the patient and prescribed two tablets of magnesium oxide 330 mg in the morning and evening to control defecation. While a patient experienced relief from constipation and urinary incontinence, his urinary frequency was not improved. The result of the blood test showed a high prostate‐specific antigen (PSA) level of 139.0 ng/ml; so, the patient and his eldest daughter were informed about the possibility of prostate cancer. Considering the patient's age, he and his eldest daughter chose to monitor his condition with regular blood tests instead of undergoing a thorough examination. In addition, the patient also began using home care nursing facilities to keep in touch with his family and to prevent deterioration of his Activities of Daily Living (ADL).

With the help of his eldest daughter, he started keeping records of his urination frequency and defecation status and managed to take care of his health condition. He used to express gratitude to his eldest daughter who was his primary caregiver. However, sometimes he expressed a rather arrogant attitude, commenting that it is an obligation of children to take care of their parents. Furthermore, despite repeated recommendations from his daughter, doctors, and support specialists, he refused to use a day‐care facility or a nursing home even for a short stay. His ADL began to decline prominently, and the frequency of urinary incontinence was increased.

As the daughter was forced to deal with his frequent urination, she was tired over time and had difficulty accepting changes in her father's condition caused by senility. In January 2019, the clinic staff received an emergency call from his family that he suddenly experienced severe back pain and became unconscious. Once the doctor visited his residence, he regained his consciousness back. The patient and family members were explained about the possibility of a sudden change in his condition, but he assured the doctor that there was no need for him to go to the hospital. Although the family wanted to fulfill his desire, they were worried about continuing the medical treatment at home and were particularly concerned about handling such emergencies.

It was hard for his daughter to balance household chores and caregiving. So, with approval from the patient, they started using a day‐care facility or a nursing home for a short stay from February 2019. There were some instances where she also had difficulty accepting the fact that her father was aging with cognitive and physical decline, so she ended up reprimanding him for his behavior. Moreover, she was worried and concerned as he refused to get admission to the hospital even in the emergency. To come up with the best possible solution, the home care team, which consisted of medical staff and care managers, discussed with him and his family about his future medical treatment plan. During the discussion, he expressed his wish to receive care from his family at home without using nursing services and to end his life at home, while his family wanted to seek help from the facility. The discussion was inconclusive, and it was difficult to come up with a final solution that could address the desire of the patient and reduce the burden of care on his family at the same time.

In June 2020, we received an emergency call with the complaint of a fever of 37.8°C and increased sputum production after breakfast. During our visit, his physical examination revealed temperature: 38.2°C, blood pressure: 162/93 mmHg, pulse: 90 beats per minute, a saturation of percutaneous oxygen: 89% at room air, the respiration rate: 26 breaths per minute, and also the presence of coarse crackles during chest auscultation. Considering such symptoms, aspiration pneumonia was suspected. If the doctor had followed his wishes at that stage, the doctor would have considered introducing home oxygen and daily antibiotic infusion by home care nurses to treat him at home. However, considering the family's condition and their inability to provide care at home, the doctor made a comprehensive judgment to take him to the hospital for emergency care. After that, he was transferred to a recovery hospital for rehabilitation. About a month later, the family informed that he had been admitted to a nursing home affiliated with the hospital and a home visit was no longer necessary.

## 3 DISCUSSION

The present case shows the difficulty for healthcare providers to become advocates for patients and ACP in the absence of robust legal backing.

In our case, although the patient's desires were understood and shared between the family and the healthcare providers, his deteriorating condition made the decision more family‐centric. There was a possibility that the lack of legal respect for the individual made it difficult to have a patient‐centered discussion.

Legislation such as AD in the United States,^3^ the Euthanasia Act in the Netherlands,^13^ and Medical Assistance in Dying (MAiD) in Canada^14^ have increased the discussion of self‐determination by legal guaranteeing the patient's right to self‐determination. However, in Japan, self‐decision is only recommended in the medical guidelines and there is no legal framework to back up such. Therefore, as in our case, when the patients' values differ from healthcare providers, and family members, it is difficult to make patient‐centric decisions. It has been reported that the participation of caregivers including family members in ACP promotes family acceptance regarding changing patient's condition and respect for patient's wishes, thereby facilitating end‐of‐life care at home.^15^, ^16^

Japanese health care has always emphasized the wishes of patients' families. Indeed, discussion of care goals between doctors and patients' families can reportedly reduce conflicts in decision‐making about patients' management plans.^17^ However, in our case, there were policy disagreements between the home care team and family members; so, it was impossible to discuss care goals and follow the patient's wishes. The healthcare providers experienced difficulty communicating with the family members, and the family members did not cooperate with ACP. This was due to the lack of a framework to reduce conflicts between the healthcare providers and the family members, suggesting the need for a legal framework to promote such discussion. In home care practice, regular patient visits provide more opportunities and time to talk to patients and their families, making it easier to advance ACP. In such a situation, having legal backing for ACP may promote the discussion of respecting patients' rights, particularly in the context of Japan.

While considering legal backing, cultural aspects also need to be taken into account. Many Japanese follow a cultural virtue of practicing “relationship‐conscious self‐decisions” because they prioritize family harmony and the result of which they want to align their personal decision with the wishes of family or group to which they belong. As a result, if the patient's wishes differ from those of others, patient is likely to suppress their wishes. In fact, family‐centered decision‐making is widely practiced in the dying stage between the doctors and the family members.^18^ Also, some patients rely heavily on the opinions of their families or leave all decisions to family members, despite their ability to make decisions on their own. Thus, given the cultural background of Japan, legal backing is important to ensure the patient's wishes are followed. As in our case, even if doctors attempt to protect the patient's wishes, there is a possibility of conflicts between the patient and the family members. However, it has been reported that decision‐making conflicts are reduced when the surrogate decision‐maker is aware of the patient's preferences.^19^, ^20^ If the ACP has legal backing in Japan and patients are enforced to make decisions on their own, it will be difficult to agree with the whole process, which may result in high stress. Thus, when discussing the legal backing in decision‐making, it is necessary to consider the cultural background of the country.

It is difficult to enact the necessary law immediately. Therefore, in order to promote ACP in this Japanese cultural and legal context, healthcare professionals need to understand the unique decision‐making style of the Japanese people as shown in this case. With the growing recognition of ACP, Japan has been increasingly emphasizing the patient's right to self‐determination. In a culture that values harmony, it is important to provide decision‐making support that prioritizes harmony of each individual patient and family unit.

## 4 CONCLUSION

In our case, we were unable to meet the wish of a patient due to the disagreement between the patient and family members regarding their choice of care facility. One of the reasons behind such was the lack of legal backing for the patient's rights. The unique relationship‐centered decision‐making practice of Japan also made it difficult to grant the patient's wishes and promote ACP effectively. Furthermore, legal assurance like those in Western countries may not always work well in Japan, but a legal framework that guarantees the rights of both patients and healthcare providers in ACP and which respects the goals and values of patients may help to promote discussions about patients' wishes in a collective culture. However, difficulties in achieving legal assurance are expected due to political and other barriers. Nevertheless, it is necessary for healthcare providers in the first place to enhance their understanding of decision‐making culture.

## CONFLICT OF INTEREST

Dr. Ozaki received a personal fee from MNES INC. outside of the submitted work.

## AUTHOR CONTRIBUTIONS

HM and AO wrote the initial draft of the manuscript. HM, AO, YK, RS, DB, YU, and HB assisted in the preparation of the manuscript. HM is the lead author and responsible for submission. All authors critically reviewed and revised the manuscript. HM and HB had full access to the data, and controlled the decision to publish, and accept full responsibility for the work.

## ETHICAL APPROVAL

This research meets the ethical guidelines and adheres to Japan's local legal requirements. An ethical review is not required for this type of article.

## CONSENT

Written informed consent was obtained from the patient's family for publication of this case report and any accompanying images.
